# Physico‐Chemical Characteristics, Antioxidant Activities, and ACE Inhibitory Potential of Pakistani Dahi Supplemented With Date Fruit Paste

**DOI:** 10.1002/fsn3.4588

**Published:** 2025-01-31

**Authors:** Tahir Mahmood Qureshi, Muhammad Nadeem, Ghulam Muhammad, Waseem Abbas, Kashif Akram, Majid Hussain, Zahid Manzoor, Salam A. Ibrahim

**Affiliations:** ^1^ Department of Food Sciences Cholistan University of Veterinary and Animal Sciences Bahawalpur Pakistan; ^2^ Institute of Food Science and Nutrition, University of Sargodha Sargodha Pakistan; ^3^ Horticultural Sciences Department, North Florida Research and Education Center Institute of Food and Agricultural Sciences, University of Florida Quincy FL USA; ^4^ Faculty of Food Science & Nutrition Bahauddin Zakariya University Multan Pakistan; ^5^ Department of Pharmacology and Toxicology Cholistan University of Veterinary and Animal Sciences Bahawalpur Pakistan; ^6^ Department of Food and Nutritional Sciences North Carolina Agricultural and Technical State University Greensboro NC USA

**Keywords:** ACE inhibition, antioxidant potential, dahi, date varieties pastes, microbiological quality, physico‐chemical characteristics

## Abstract

Dahi as well as date fruits are considered very important in our diet due to the presence of nutritional and medicinal properties. The objective of this study was to evaluate the antioxidant potential and angiotensin‐I‐converting enzyme (ACE) inhibitory activity of date paste added to indigenous dahi during storage (0–8 days, 5°C). The prepared dahi was also evaluated for its physico‐chemical characteristics and microbiological and sensorial quality during the storage period. The freshly prepared dahi supplemented (prepared from both cow's and buffalo's milk) with different date fruit pastes showed less syneresis, improved water holding capacity as well as sensorial quality compared to the control. The freshly prepared buffalo's milk dahi supplemented with different date fruit pastes performed the best with regard to TP contents (0.54–0.89 mg gallic acid equivalent/g), TF contents (163.43–243.42 mg quercetin equivalent/100 g), FRAP (733.11–898.66 μg ascorbic acid equivalent/g), and TAC (164.30–254.10 mg ascorbic acid equivalent/100 g) compared to the control. Regarding ACE inhibition, the freshly prepared buffalo's milk dahi supplemented with date paste showed higher values (61.36%–72.43%) than the control (49.03%). The dahi prepared from buffalo's milk exhibited improved physico‐chemical characteristics, antioxidant as well as ACE inhibition (%) than those of cow's milk dahi. It was concluded that dahi supplemented with date pastes from different varieties exhibited improved physico‐chemical characteristics, antioxidant activity as well as ACE inhibition (%) than those of control dahi.

## Graphical abstract

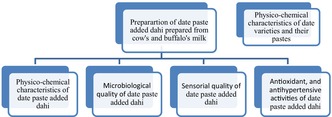

## 1 Introduction

Yogurt is a fermented dairy product that is produced as a result of the inoculation of pure starter cultures (*Streptococcus thermophiles*, *Lactobacillus delbrueckii* spp. *bulgaricus*) (Selvamuthukumaran and Farhath 2014; Mohamed, Zayan, and Shahein 2014). Yogurt has been a part of the human diet for several millennia and is known by different names in different cultures. The Indian ayurvedic scripts (around 6000 BCE) referred to the health benefits of fermented milk products (Fisberg and Machado 2015). For example, yogurt is considered to be an excellent source of highly bioavailable protein and probiotics that may provide many health benefits (Fisberg and Machado 2015). In Pakistan, India, Nepal, and Bangladesh, yogurt is synonymously known as dahi. Dahi can be included in any meal but is typically consumed at breakfast after being sweetened with sugar. Traditionally, in Pakistan, pure starter cultures are not used in milk for making fermented dairy products. Instead, the back‐slopping technique is traditionally used for making dahi. With back‐slopping, a very small portion of already prepared dahi is used in milk for setting dahi. The predominant bacterial cultures present in dahi include the same starter bacteria (*Streptococcus thermophiles*, *Lactobacillus delbrueckii* spp. *bulgaricus*) that are used for making yogurt. In addition to the abovementioned predominant bacterial cultures, many other bacteria are also present in dahi. Owing to the presence of common bacterial cultures, both dahi and yogurt have similar physico‐chemical characteristics; however, the sugar that is typically added to dahi can be replaced by natural date fruit paste. Consequently, the deleterious effects of sugar can be avoided and the benefits of dates can be achieved.

Traditionally, dahi is thought to prevent digestive disorders and to aid in the development of bones. Various studies regarding the physico‐chemical and microbiological characteristics of dahi have been conducted in Bangladesh, India, and Nepal (Afrin et al. 2016; Biswas et al. 2017; Bhattarai and Das 2015). Some exopolysaccharide‐producing *Streptococcus thermophilus* strains have also been screened from dahi for their probiotic potential (Taj et al. 2022). Farid et al. (2021) also investigated *Lactobacillus acidophilus* strains from dahi for their excellent properties of antioxidants and exopolysaccharide production. Moreover, the antioxidant potential of yogurt supplemented with different fruits has been explored (reviewed by Qureshi et al. 2017).

Date palm fruit is commonly consumed in the Middle East and around the world due to its nutritional and medicinal properties as well as its economic success. The antioxidant activity and phenolic contents of some date fruit cultivars grown in Saudi Arabia, Oman, the United States, Iran, Tunisia, and Algeria have already been investigated (Al‐Juhaimi, Ghafoor, and Özcan 2014; Al‐Farsi et al. 2005; Al‐Turki, Shahba, and Stushnoff 2010; Biglari, AlKarkhi, and Easa 2008; Chaira et al. 2007; Ghiaba et al. 2014). Similarly, the antioxidant activity and phenolic contents of some date varieties from Pakistan have been reported (Nadeem, Anjum, and Bhatti 2011; Haider et al. 2014).

Hypertension is a ubiquitous health concern in Pakistan and globally. This is primarily due to the presence of increased saturated fats in daily diets. The angiotensin‐I‐converting enzyme (ACE) inhibitory activity of food and its constituents is related to antihypertensive activity. It has been reported that there is a strong association between dietary intake of saturated fats and hypertension (Hassana et al. 2019; Gou et al. 2022). Therefore, people suffering from hypertension and obesity should thus avoid food products that contain considerable saturated fat content and instead focus on products having negligible quantities of saturated fats. In this regard, owing to the health aspects of milk (having negligible quantities of fats) and date fruit, low‐fat dahi supplemented with date paste would be a wiser choice. In addition, there is a variation between cow's milk and buffalo's milk composition. The quality of dahi prepared from buffalos' milk and cow's milk may vary due to variations in the milk constituents. As a result, our research team felt that it would be interesting to observe quality variations in dahi using both cow's milk and buffalo's milk.

Previously, date fruits or its paste have been valorized into many products, such as cookies, sandwich spread, and snack bars (Ibrahim et al. 2021, 2020). To date and to our knowledge, no study has been published regarding monitoring the antioxidant potential and antihypertensive activity of low‐fat dahi (using both cow's and buffalo's milk) supplemented with date fruit paste. Thus, the objective of the present study was to examine the impact of date fruit paste on the physico‐chemical characteristics, antioxidant activities, and ACE inhibition of low‐fat dahi, a traditional yogurt product in Pakistan.

## 2 Material and Methods

### 2.1 Chemicals and Equipments

DPPH (2, 2‐diphenyl‐1‐picrylhydrazyl), ascorbic acid, Folin–Ciocalteu reagent, Catechin, Trolox, and many other chemicals/standards were purchased from Merck (KGaA, Darmstadt, Germany). Plate count agar media was purchased from acumedia, LAB, Neogen Culture Media, USA whereas potato dextrose agar was purchased from Biolife, Milano, Italia. Sodium nitrite, aluminum chloride, potassium ferricyanide, trichloroacetic acid, ferric chloride, potassium persulfate, potassium phosphate, 4 mM ammonium molybdate, NaCl, etc. were also of analytical grade.

### 2.2 Procurement of Milk and Date Fruits

Cow' milk and buffalo's milk were collected from a local dairy farm at Bahawalpur city and ripened date fruit cultivars, that is, Muzafati, Popo, Aseel, and Ajwa were brought from the local market to the Department of Food Sciences, Cholistan University Veterinary and Animal Sciences, Bahawalpur.

### 2.3 Preparation of Date Paste

All the date varieties (Muzafati, Popo, Aseel, and Ajwa) were cut into small pieces after depitting and thorough washing. They were steamed (95°C for 15 min) for inactivation of any enzyme and to make the flesh soft. The peels were separated using hands and the flesh part was made homogenous with the help of mortar and pestle to make paste.

### 2.4 Physico‐Chemical Analysis of Milk, Date Fruits, and Their Pastes

The pH of milk was monitored using a pH meter after calibrating with buffer of pH 4.0 and pH 7.0. The fat in milk was determined by the Gerber‐van Gulik method using a butyrometer after standardization. Total protein (%) of milk was estimated according to the Association of Official Analytical Chemists (AOAC 2012). Lactometer was used to calculate solids‐not‐fat (SNF%) of milk.

The weight (g) of each date fruit (variety) was measured using an analytical balance (Electronic scale JJ224BC; Mettler Toledo, USA). The date fruits were also investigated for moisture, protein, ash, fat, and fiber contents (AOAC 2012). The carbohydrate contents of investigated date fruit varieties were determined by subtracting the moisture, protein, ash, fat, and fiber contents from the total mass (i.e., 100). The energy values of date varieties were also calculated by summing the multiplied values for protein, fat, and carbohydrate by their respective factors (4, 9, and 4).

Lane and Eynon method (AOAC 2012) was employed to determine total sugars, reducing sugars, and non‐reducing sugars. Ten (10 g) grams of date paste from each variety were added into 100 mL of distilled water. Then, 10 g of citric acid was added to the mixture and boiled for 8 min for inversion of sugars. The mixture was neutralized with NaOH after cooling to room temperature and diluted up to 200 mL with distilled water. The prepared filtered (sample) solution was filled in the burette and was added into 10 mL Fehling's solution (Fehling A (copper sulfate (69.28 g per 1000 mL distilled water)) and Fehling B (346 g Rochelle salt (potassium sodium tartarate)), and 100 g sodium hydroxide per 1000 mL distilled water). The Fehling solution is heated and boiled continuously on the hot plate while sample solution is added. After Fehling solution just started to boil, three to four drops of methylene blue were also added immediately. The sample solution from the burette (1 mL each time after an interval of 10 s) was added to the boiled mixture until it is turned into brick red color. The titer values were corresponded to the total reducing sugar contents (mg/100 mL) as calculated with the formula (B). Same procedure was followed to find out reducing sugars (A); however, in this process date paste mixture is not boiled with citric acid for inversion of sugars. Reducing, non‐reducing, and total sugars were calculated with the formulae given below.

$$\text{Reducing sugars}\;\left(\%\right)\;\text{before inversion}\;\left(A\right)=\frac{\text{Factor}\times \text{Dilution}\;}{\text{Titer value}}\times 100$$

$$\text{Reducing sugars}\;\left(\%\right)\;\text{after inversion}\;\left(B\right)=\frac{\text{Factor}\times \text{Dilution}\;}{\text{Titer value}}\times 100$$

$$\text{Non}-\text{reducing sugars}\;\left(C\right)=\left(B–A\right)$$

Total sugars=A+C

Vitamin C (ascorbic acid) contents of date paste from each variety were also determined (AOAC, 2012). Date paste (10 g) was homogenized with 90 mL of 3% metaphosphoric acid (HPO_3_) and centrifuged at 9000 g for 15 min. The obtained clear supernatant was titrated against 2, 6‐dichlorophenol indophenol dye, till the pink rose color persists for about 20 s. The titration was also carried out with ascorbic acid (1%) solution in the same manner and the results were calculated as mg ascorbic acid 100 g^−1^ sample.

### 2.5 Production of Dahi Supplemented With Date Paste

Dahi was produced according to the method as described by Perna et al. (2014) with some modifications. In our preliminary trials, date paste were mixed with milk at different ratios but finally, 10:90 ratio was selected for date paste and milk for all the treatments based on better sensorial quality of the final products. In this way, the blend (5 L) of each date paste and milk (each treatment) was pasteurized at 82°C for 5 min and cooled to 45°C for back‐slopping (inoculation by already prepared normal dahi). The incubation was done for 3–4 h at 42°C until the pH reached 4.6. Control (dahi) treatment (only milk without addition of date paste) was designated as T_0_. The treatment denoted as T_1_ was the dahi prepared from the mixture of Muzafati paste and milk whereas T_2_ was the treatment prepared from the mixture of Popo paste and milk. The treatment denoted as T_3_ was the dahi prepared from the mixture of Aseel paste and milk whereas T_4_ was the treatment prepared from the mixture of Ajwa paste and milk (Table 1). The dahi samples were stored for 2, 4, 6, and 8 days at refrigerated temperature. Fresh dahi (0 day) was also analyzed. The production of dahi samples was done in three batches.

**TABLE 1:** Treatment plan of the present study.

Treatments	Date fruit paste	Milk
Control (dahi) treatment (T_0_)	—	100%
Muzafati paste dahi (T_1_)	10%	90%
Popo paste dahi (T_2_)	10%	90%
Aseel paste dahi (T_3_)	10%	90%
Ajwa paste dahi (T_4_)	10%	90%

### 2.6 Physico‐Chemical Characteristics of Prepared Dahi

The pH of dahi was monitored using a pH meter. Acidity of dahi was determined by AOAC (2012). Moisture contents of dahi were determined according to AOAC (2012). The fat contents of dahi were determined by the Gerber‐van Gulik method. Total protein (%) and ash (%) of dahi were estimated according to AOAC (2012).

### 2.7 Microbiological Analysis of Prepared Dahi

Dahi was microbiologically analyzed for total plat counts (TPC, log CFU/g of dahi) and yeast, and mold (Y&M, log CFU/g of dahi) during storage period by following method as described by Broadbent et al. (2013). Ten (10 g) grams dahi was homogenized with 90 mL of sterilized sodium citrate (2%, pH 7.5) water. One milliliter of different dilutions (up to 10^−4^) of the above mixture was used on plates after pouring plate count agar media. The petri plates were incubated for 2 days (37°C) for the enumeration of TPC. In the same manner, perti‐plates were prepared and the counts of Y&M were carried out after 48 h at 30°C by using potato dextrose agar.

### 2.8 Sensory Evaluation of Prepared Dahi

Dahi was evaluated for its appearance and color, flavor, texture, and overall acceptability using a 9‐point hedonic scale (1 = dislike extremely, 2 = dislike very much, 3 = dislike moderately, 4 = dislike slightly 5 = neither like nor dislike, 6 = like slightly, 7 = like moderately, 8 = like very much, and 9 = like extremely) as described by Korkmaz, Bilici, and Korkmaz (2021). The sensory characteristics of prepared dahi samples were assessed by 10 panelists (22–28 age, males and females). The group consisted of graduates from the Department of Food Sciences at our university who know about the scaling procedures of dahi samples and consume dahi regularly in their diets.

### 2.9 Syneresis (%) and Water Holding Capacity (%) of Dahi

Syneresis (%) and water holding capacity (WHC) (%) of dahi samples prepared in the present study were evaluated by the following methods described by Korkmaz, Bilici, and Korkmaz (2021). For syneresis, 100 mL of dahi was filtered through Whatman No. 1 for 6 h. The volume of the obtained whey was calculated using the following formula:

$$\text{Syneresis}\;\left(\%\right)=\left(\text{Whey volume}/\text{Initial volume}\right)\times 100$$

In order to analyze WHC of dahi, 5 g sample was centrifuged (5000 rpm, 15 min, 4°C). The whey obtained was weighed and the WHC was calculated using the following formula:

$$\mathrm{WHC}\;\left(\%\right)=\left[1–\left(\text{Whey weight}/\text{initial weight}\right)\right]\times 100$$

### 2.10 Preparation of Water Soluble Extract of Dahi

The procedure described by Gupta et al. (2013) with some modifications was adapted for the preparation of water soluble extract (WSE) of dahi. Briefly, dahi was centrifuged (Hermle Labortechnik GmbH Siemensstr‐25 D‐78564 Wehingen, Germany) at 14,000 ***g*** for 10 min at 4°C. The supernatant was denoted as WSE which was filtered through a Whatman No.1 filter paper. All the extracts were immediately frozen at −20°C.

### 2.11 Total Phenolic Contents

The total phenolic (TP) contents of WSE of dahi were determined by using Folin–Ciocalteu reagent method with some modifications (Reis et al. 2012). Folin–Ciocalteu reagent (5%, 1500 μL) was added into WSE (500 μL) of dahi. Then, 1500 μL of sodium carbonate (10%) solution was added into the mixture. The absorbance was taken at 760 nm using a UV/VIS Spectrophotometer (T80, PG Instruments) after an incubation of the mixture for 60 min in dark. The TP contents were calculated as μg gallic acid equivalent (GAE)/g of dahi.

### 2.12 Total Flavonoid Contents

The total flavonoid (TF) contents of dahi were also determined by the spectrophotometric method as described by Jia, Mengcheng, and Jianming (1999). Sodium nitrite (5%, 75 μL) solution was mixed with WSE (500 μL) of dahi. Then, aluminum chloride (10%, 150 μL) solution was added into the mixture. After addition of 500 μL of NaOH (1 M) into the above mixture, the absorbance was taken at 510 nm using UV/VIS Spectrophotometer (T80, PG Instruments). The results were articulated as μg quercetin equivalent (QE)/g of dahi.

### 2.13 Ferric Reducing Antioxidant Power Assay

The ferric reducing antioxidant power (FRAP) of WSE of dahi was determined using the method as described by Reis et al. (2012) with some modifications. The sample (500 μL WSE of dahi) was mixed with 500 μL each potassium phosphate buffer (0.2 M, pH 6.6) and potassium ferricyanide (K3Fe(CN6)) in sequence. Then, 500 μL of trichloroacetic acid (%) was added into the above mixture after incubation for 20 min at 50°C. The mixture was centrifuged (Hermle Labortechnik GmbH, Wehingen, Germany) at 5000 rpm (10 min at 4°C) for clear supernatant. Then, 200 μL of ferric chloride (0.2%) was finally added to the supernatant and the absorbance was taken by using UV/VIS Spectrophotometer (T80, PG Instruments, UK) at 700 nm. The results were calculated as μg ascorbic acid equivalent (AAE)/g dahi.

### 2.14 ABTS Radical Scavenging Activity Assay

The capability of date paste added dahi to scavenge 2,2′‐azino‐bis(3‐ethylbenzothiazoline‐6‐sulfonic acid) diammonium salt (ABTS) was determined according to the method as described by Zeghad *et al*. (2019). Potassium persulfate (2.5 mM) was mixed with 7 mM ABTS solution (1:1 in distilled water) and allowed to stand in the dark for 20 h. The mixture was then diluted using ethanol so as to adjust the absorbance of 0.70 ± 0.02 at 734 nm. For, 30 μL (each sample or standard solution) was added into 3 mL of ABTS solution at room temperature. The absorbance of total mixture was taken after 6 min. The ABTS radical scavenging activity was calculated as μmol ascorbic acid equivalent (AAE) per g.

### 2.15 DPPH Radical Scavenging Activity Assay

The capability of WSE of dahi to scavenge 2, 2‐diphenyl‐1‐picrylhydrazyl radical (DPPH˙) was determined according to the method of Yi et al. (2008) with some modifications. One (1) mL WSE of dahi was mixed with 2 mL DPPH (60 μM) solution. The mixture was placed in the dark for 30 min. The absorbance was taken at 517 nm by using UV/VIS Spectrophotometer (T80, PG Instruments, UK). The DPPH radical scavenging activity was calculated as μmol ascorbic acid equivalent (AAE) per g.

### 2.16 Total Antioxidant Capacity Assay

The method described by Prieto, Pineda, and Aguilar (1999) was used for the determination of total antioxidant capacity (TAC) of dahi. One (1) mL WSE of dahi was mixed with 3 mL of reagent (28 mM of potassium phosphate, 0.6 M sulfuric acid, and 4 mM ammonium molybdate) solution. After incubation (95 min at 90°C) of the mixture, the absorbance was taken at 695 nm using a UV/VIS Spectrophotometer (T80, PG Instruments, UK). The TAC was calculated as mg ascorbic acid equivalent (AAE) per 100 g.

### 2.17 ACE Inhibitory Activity

The ACE inhibitory activity of WSE of dahi was measured using the spectrophotometric assay as described by Ong and Shah (2008). Hippuryl–histidyl–leucine (HHL, Sigma, 5 mM) was mixed in potassium phosphate buffer (0.1 M, pH 8.3) having NaCl (0.4 M). The extract from rabbit lung acetone powder was prepared by using the method of Vermeirssen, Van‐Camp, and Verstraete (2002). A mixture of sample (25 μL) and HHL solution (225 μL) was incubated (37°C, 5 min). Then, incubation was done (30 min) after addition of 75 μL ACE solution (rabbit lung acetone powder extract). The reaction was stopped with 20 μL of HCl (5 M). The mixture was centrifuged (5000 g, 10 min) after the addition of ethyl acetate and 1.5 mL of ethyl acetate phase was transferred to a fresh test tube and evaporated to dryness on a water bath. The residues having hippuric acid were dissolved in 1 mL distilled water and the absorbance was taken using a UV visible spectrophotometer at 228 nm against distilled water as a blank. The percent inhibition was calculated as follows: ACE inhibition (%) = [1 – (A–C)/(B–D)] × 100, where A is the abs. with sample, ACE and HHL, B is the abs. with ACE and HHL without sample, C is the abs. with sample and HHL (without ACE), and D is the abs. with HHL without sample and ACE.

### 2.18 Statistical Analysis

Statistical analysis was performed through Minitab statistical software version 16 (Minitab Inc., State College, PA, USA), using the general linear model and Tukey's test for pairwise comparison in analysis of variance at the level of *p <* 0.05. Moreover, Pearson's correlation coefficient (R) (*p* value) between the investigated antioxidant capacity parameters was also calculated.

## 3 Results And Discussion

### 3.1 Physico‐Chemical Characteristics of Milk and Date Varieties

The pH of cow's milk was 6.8 whereas buffalo's milk pH was 6.7. The fat in cow's milk as well as buffalo's milk was standardized at 2%. Total protein (%) of cow's milk was 3% while buffalo's milk showed 4.7%. The SNF (%) of cow's milk were 8.6 whereas buffalo's milk was found to have 10.2%.

Table 2 presents the data regarding physico‐chemical characteristics of the investigated date varieties. Moisture, ash, crude fat, crude fiber, total protein, carbohydrate content, estimated energy values, and acidity showed significant (*p <* 0.05) variations between date varieties. The highest fruit weight (10.17 g) was shown by Popo. The highest pH value (5.48) and moisture contents (26.14%) were shown by Muzafati. The highest contents of ash (4.01%) and fiber (2.48%) were shown by Ajwa. Ajwa also showed minimum value of acidity (0.59%). There were non‐significant (*p* < 0.05) variations of carbohydrates and energy values between Popo, Aseel, and Ajwa. These energy values are attributed to the contents of carbohydrate. All the date varieties contained very low content of protein, fat, and fiber. In addition, Popo and Ajwa dates had lower acidity contents compared to other investigated date fruits. Among the investigated date varieties, Muzafati dates had soft nature due to presence of more moisture whereas Popo and Ajwa dates were little bit dry as depicted from their moisture contents.

**TABLE 2:** Physico‐chemical characteristics of date varieties.

Properties	Date varieties			
	Muzafati	Popo	Aseel	Ajwa
Moisture (%)	26.14 ± 1.65a	17.50 ± 0.95c	21.00 ± 1.68b	17.00 ± 0.50c
Fruit weight (g)	7.03 ± 0.71b	10.17 ± 0.76a	5.21 ± 0.09c	7.47 ± 0.29b
Ash (%)	3.09 ± 0.07c	3.53 ± 0.07b	2.51 ± 0.10d	4.01 ± 0.21a
Protein (%)	1.01 ± 0.06c	1.21 ± 0.07b	0.80 ± 0.05d	2.09 ± 0.08a
Fat (%)	0.80 ± 0.06b	0.59 ± 0.05c	1.20 ± 0.05a	0.40 ± 0.04d
Fiber (%)	1.27 ± 0.07c	2.10 ± 0.07b	0.55 ± 0.05d	2.48 ± 0.07a
Carbohydrates (%)	68.68 ± 1.04b	76.04 ± 1.04a	74.93 ± 1.50a	75.01 ± 0.50a
Energy (kcal/100 g)	285.99 ± 6.7b	314.40 ± 4.08a	313.72 ± 7.03a	312.03 ± 2.90a
pH	5.48 ± 0.12a	4.58 ± 0.06c	5.43 ± 0.13a	5.00 ± 0.07b
Acidity (%)	1.15 ± 0.05a	1.02 ± 0.04b	1.22 ± 0.07a	0.59 ± 0.02c

*Note:* Means with different letters in the same row showed significant (*p <* 0.05) variations between different date varieties.

### 3.2 Physico‐Chemical Characteristics of Date Paste

Table 3 shows pH, acidity, vitamin C, total sugars, reducing sugars, and non‐reducing sugars of date paste from all varieties. There were significant (*p <* 0.05) variations in the values of pH and acidity between pastes from different date varieties. The maximum (6.90) pH was observed in Aseel paste (6.90) whereas minimum (6.30) was found in Ajwa paste. The minimum (0.75%) acidity was noted in Ajwa paste whereas maximum (1.17%) was observed in muzafati paste. Considerable quantities of vitamin C were observed in the pastes from Ajwa (1.47 mg/100 g) and Muzafati (1.40 mg/100 g). The maximum total sugars were found in Muzafati paste (55.51%) followed by Aseel (53.05%) and Ajwa paste (49.64%). Non‐reducing sugars were present in very less quantities in all the date pastes as compared to the reducing sugar contents. The highest acidity, total sugars, and reducing sugar contents were present in Muzafati paste. The lowest acidity and the highest contents of vitamin C were present in Ajwa paste.

**TABLE 3:** pH, acidity, vitamin C, total sugars, reducing sugars, and non‐reducing sugars of date paste.

Date varieties	pH	Acidity (%)	Vitamin C^a^	Total sugars (%)	Reducing sugars (%)	Non‐reducing sugars (%)
Muzafati paste	6.51 ± 0.04b	1.17 ± 0.03a	1.40 ± 0.04a	55.51 ± 1.98a	53.02 ± 0.60a	2.19 ± 0.55b
Popo paste	6.42 ± 0.02c	0.81 ± 0.02c	0.67 ± 0.03c	46.47 ± 2.13c	40.80 ± 0.77c	5.67 ± 0.52a
Aseel paste	6.90 ± 0.03a	0.92 ± 0.03b	0.80 ± 0.05b	53.05 ± 2.18a	48.40 ± 1.05b	4.65 ± 0.66a
Ajwa paste	6.30 ± 0.03d	0.75 ± 0.02c	1.47 ± 0.03a	49.64 ± 1.92b	47.97 ± 0.35b	1.87 ± 0.42b

*Note:* Means with different letters in the same column show significant (*p <* 0.05) differences.

^a^ mg/100 g of date paste.

### 3.3 Physico‐Chemical Characteristics of Prepared Dahi

Table 4 shows the effect of date fruit paste addition on proximal composition of all dahi samples (treatments) prepared from cow's milk. The pH of control cow's milk dahi (without the addition of date paste) started with 4.48 which continued to drop reaching till 4.22 after 8 days of storage. But dahi samples supplemented with date paste showed slightly lower values (~4.40) compared to control paneer in the start. The pH values of date paste added dahi dropped too much extent during storage reaching until ~4.05. Moisture contents of all dahi samples were very slightly decreased during storage. Control dahi was found to have slightly higher moisture contents (~87%) compared to all other date paste added dahi. The TS of all the samples did not show significant variations during storage. The TS of control dahi were lower (~12.50) compared to the values of dahi supplemented with date pastes (13%–16%). In general, pH and moisture of control dahi were higher than date paste supplemented dahi whereas acidity, fat, ash, and TS of date paste added dahi were higher than control dahi.

**TABLE 4:** Physico‐chemical characteristics (means ± SD) of cow's milk dahi (T_0_ = control, T_1_ = Muzafati paste dahi, T_2_ = Popo paste dahi, T_3_ = Aseel paste dahi, T_4_ = Ajwa paste dahi) supplemented with paste from different date varieties during storage.

Treatments	Days	pH	Acidity (%)	Moisture(%)	Fat (%)	Protein (%)	Ash (%)	TS	Fat/TS
T_0_	0	4.48 ± 0.07a	0.63 ± 0.05gh	87.46 ± 0.67a	1.80 ± 0.10d	4.00 ± 0.76a	0.90 ± 0.08a	12.53 ± 0.67h	14.40 ± 1.40i
	2	4.40 ± 0.04a	0.77 ± 0.09c–h	87.76 ± 0.67a	1.82 ± 0.04d	3.97 ± 0.49a	0.94 ± 0.10a	12.23 ± 0.67h	14.91 ± 1.04g–i
	4	4.30 ± 0.04a–d	0.83 ± 0.08b–h	87.20 ± 0.60ab	1.86 ± 0.08d	4.04 ± 0.35a	0.96 ± 0.01a	12.79 ± 0.60gh	14.56 ± 1.16hi
	6	4.30 ± 0.04a–d	0.96 ± 0.11a–e	87.83 ± 0.23a	1.85 ± 0.03d	4.08 ± 0.49a	0.97 ± 0.15a	12.16 ± 0.23h	15.20 ± 0.08e–i
	8	4.22 ± 0.03a–d	1.02 ± 0.10a–d	87.62 ± 0.50a	1.87 ± 0.04d	4.09 ± 0.72a	0.99 ± 0.21a	12.37 ± 0.50h	15.12 ± 0.63f–i
T_1_	0	4.39 ± 0.06a	0.62 ± 0.04gh	86.43 ± 0.13a–c	2.70 ± 0.11bc	4.40 ± 0.40a	1.31 ± 0.90a	13.56 ± 0.13f–h	19.90 ± 0.92a–c
	2	4.30 ± 0.06a–d	0.81 ± 0.06c–h	85.50 ± 0.61c–f	2.80 ± 0.08a–c	4.44 ± 0.25a	1.34 ± 0.07a	14.49 ± 0.61b–f	19.32 ± 0.27a–d
	4	4.20 ± 0.05a–d	0.94 ± 0.07a–f	85.36 ± 0.29c–g	2.85 ± 0.05a–c	4.42 ± 0.55a	1.36 ± 0.10a	14.63 ± 0.29b–f	19.47 ± 0.56a–d
	6	4.19 ± 0.04a–d	0.97 ± 0.10a–e	85.34 ± 0.20c–h	2.90 ± 0.04a–c	4.49 ± 0.30a	1.38 ± 0.14a	14.66 ± 0.20a–f	19.78 ± 0.54a–d
	8	4.01 ± 0.01d	1.13 ± 0.14ab	85.63 ± 0.25c–e	2.94 ± 0.06ab	4.48 ± 0.40a	1.40 ± 0.13a	14.36 ± 0.25d–f	20.46 ± 0.12ab
T_2_	0	4.40 ± 0.07a	0.55 ± 0.06h	84.96 ± 0.66c–h	2.74 ± 0.05a–c	4.53 ± 0.15a	1.49 ± 0.02a	15.03 ± 0.66a–f	18.25 ± 0.95b–d
	2	4.30 ± 0.01a–d	0.67 ± 0.05d–h	84.85 ± 0.15d–h	2.83 ± 0.06a–c	4.55 ± 0.70a	1.53 ± 0.05a	15.14 ± 0.15a–e	18.69 ± 0.50a–d
	4	4.27 ± 0.02a–d	0.78 ± 0.08c–h	84.23 ± 0.23e–h	2.94 ± 0.04ab	4.40 ± 0.35a	1.56 ± 0.06a	15.76 ± 0.23a–d	18.82 ± 0.51a–d
	6	4.20 ± 0.08a–d	0.89 ± 0.09a–g	84.33 ± 0.27e–h	2.95 ± 0.08ab	4.59 ± 0.60a	1.59 ± 0.04a	15.66 ± 0.27a–d	18.78 ± 0.23a–d
	8	4.06 ± 0.06b–d	1.06 ± 0.13a–c	84.34 ± 0.38e–h	2.94 ± 0.05ab	4.63 ± 0.50a	1.62 ± 0.03a	15.65 ± 0.38a–d	18.78 ± 0.34a–d
T_3_	0	4.37 ± 0.07ab	0.65 ± 0.05f–h	83.89 ± 0.73gh	2.72 ± 0.05bc	4.60 ± 0.28a	1.28 ± 0.09a	16.11 ± 0.73ab	16.90 ± 0.84d–i
	2	4.30 ± 0.04a–d	0.78 ± 0.09c–h	83.98 ± 0.83f–h	2.87 ± 0.06a–c	4.55 ± 0.60a	1.30 ± 0.07a	16.01 ± 0.83a–c	17.96 ± 1.25b–f
	4	4.26 ± 0.01a–d	0.85 ± 0.12a–h	83.83 ± 0.65h	2.84 ± 0.04a–c	4.59 ± 0.55a	1.32 ± 0.10a	16.17 ± 0.65a	17.58 ± 0.91b–g
	6	4.22 ± 0.05a–d	0.97 ± 0.18a–e	84.24 ± 0.26e–h	2.75 ± 0.08a–c	4.63 ± 0.75a	1.33 ± 0.14a	15.75 ± 0.26a–d	17.45 ± 0.26c–h
	8	4.03 ± 0.05d	1.05 ± 0.14a–d	84.43 ± 0.45d–h	2.64 ± 0.05c	4.66 ± 0.20a	1.30 ± 0.13a	15.56 ± 0.45a–e	16.96 ± 0.21c–i
T_4_	0	4.43 ± 0.10a	0.61 ± 0.04gh	84.67 ± 0.45d–h	2.76 ± 0.16a–c	4.60 ± 0.26a	1.34 ± 0.90a	15.33 ± 0.45a–e	18.07 ± 1.53b–e
	2	4.46 ± 0.29a	0.75 ± 0.06d–h	84.23 ± 0.20e–h	2.90 ± 0.19a–c	4.51 ± 0.24a	1.37 ± 0.09a	15.76 ± 0.20a–d	18.39 ± 1.29a–d
	4	4.36 ± 0.29a–c	0.89 ± 0.07a–g	84.76 ± 0.29d–h	2.87 ± 0.06a–c	4.59 ± 0.37a	1.39 ± 0.12a	15.23 ± 0.29a–e	18.86 ± 0.41a–d
	6	4.22 ± 0.07a–d	0.99 ± 0.10a–d	84.64 ± 0.46d–h	3.00 ± 0.18a	4.56 ± 0.28a	1.41 ± 0.15a	15.35 ± 0.46a–e	19.56 ± 1.60a–d
	8	4.05 ± 0.08cd	1.15 ± 0.14a	85.86 ± 0.69b–d	3.00 ± 0.13a	4.61 ± 0.35a	1.43 ± 0.14a	14.13 ± 0.69e–g	21.33 ± 1.97a

*Note:* Means with different letters in the same column show significant (*p <* 0.05) differences between treatments and storage period.

Table 5 shows the effect of date fruit paste addition on proximal composition of all dahi samples (treatments) prepared from buffalo's milk. The pH of dahi prepared from buffalo's milk was slightly higher than dahi made from cow's milk. The moisture contents of dahi prepared from buffalo's milk was lower than dahi made from cow's milk. The more TS of buffalo's milk resulted in less moisture contents. Similarly, protein (%), ash and TS of dahi prepared from buffalo's milk were more compared to dahi made from cow's milk. Among all the treatments (dahi from cow's and buffalo's milk dahi), there was an increasing trend of acidity during storage while decreasing trend was observed concerning pH.

**TABLE 5:** Physico‐chemical characteristics (means ± SD) of buffalo's milk dahi (T_0_ = control, T_1_ = Muzafati paste dahi, T_2_ = Popo paste dahi, T_3_ = Aseel paste dahi, T_4_ = Ajwa paste dahi) supplemented with paste from different date varieties during storage.

Treatments	Days	pH	Acidity (%)	Moisture(%)	Fat (%)	Protein (%)	Ash (%)	TS	Fat/TS
T_0_	0	4.38 ± 0.07a	0.53 ± 0.05e–h	84.46 ± 0.67a	2.00 ± 0.10e	5.50 ± 0.76a	1.20 ± 0.08a	15.53 ± 0.67d	12.90 ± 1.07e
	2	4.28 ± 0.05ab	0.59 ± 0.09c–h	84.36 ± 0.67a	2.02 ± 0.04e	5.47 ± 0.49a	1.25 ± 0.10a	15.63 ± 0.67d	12.98 ± 0.69de
	4	4.17 ± 0.06a–d	0.64 ± 0.08c–h	84.16 ± 0.65ab	2.06 ± 0.08e	5.54 ± 0.35a	1.28 ± 0.05a	15.83 ± 0.65cd	13.07 ± 0.87de
	6	4.15 ± 0.06a–d	0.79 ± 0.11a–g	84.56 ± 0.36a	2.06 ± 0.03e	5.58 ± 0.49a	1.30 ± 0.16a	15.43 ± 0.36d	13.35 ± 0.37c–e
	8	4.06 ± 0.07b–d	0.85 ± 0.10a–d	84.45 ± 0.34a	2.08 ± 0.05e	5.59 ± 0.72a	1.31 ± 0.21a	15.54 ± 0.34d	13.40 ± 0.42c–e
T_1_	0	4.29 ± 0.06ab	0.52 ± 0.04e–h	80.96 ± 0.81cd	2.90 ± 0.11cd	5.90 ± 0.40a	1.61 ± 0.90a	19.03 ± 0.81ab	15.26 ± 1.19b–e
	2	4.18 ± 0.07a–c	0.63 ± 0.06c–h	80.86 ± 0.80cd	3.00 ± 0.08a–d	5.94 ± 0.25a	1.65 ± 0.08a	19.13 ± 0.80ab	15.74 ± 1.06a–e
	4	4.07 ± 0.06b–d	0.75 ± 0.07a–h	80.69 ± 1.09cd	3.05 ± 0.05a–d	5.92 ± 0.55a	1.68 ± 0.11a	19.30 ± 1.09ab	15.87 ± 1.14a–e
	6	4.06 ± 0.06b–d	0.80 ± 0.10a–f	80.60 ± 0.76cd	3.11 ± 0.04a–d	5.99 ± 0.30a	1.71 ± 0.15a	19.39 ± 0.76ab	16.05 ± 0.83a–e
	8	3.87 ± 0.06d	0.96 ± 0.14ab	80.26 ± 1.29cd	3.15 ± 0.06a–c	5.98 ± 0.40a	1.72 ± 0.14a	19.73 ± 1.29ab	16.03 ± 1.34a–e
T_2_	0	4.30 ± 0.07ab	0.45 ± 0.06h	80.36 ± 1.21cd	2.94 ± 0.05a–d	6.03 ± 0.15a	1.79 ± 0.02a	19.63 ± 1.21ab	15.02 ± 1.16b–e
	2	4.18 ± 0.03a–c	0.49 ± 0.05gh	81.02 ± 0.80cd	3.03 ± 0.06a–d	6.05 ± 0.70a	1.84 ± 0.06a	18.98 ± 0.80ab	16.01 ± 0.47a–e
	4	4.14 ± 0.05a–d	0.59 ± 0.08c–h	80.10 ± 1.40cd	3.14 ± 0.04a–d	5.90 ± 0.35a	1.88 ± 0.08a	19.90 ± 1.40ab	15.87 ± 1.32a–e
	6	4.07 ± 0.09b–d	0.72 ± 0.09a–h	80.03 ± 0.60cd	3.16 ± 0.08a–c	6.09 ± 0.60a	1.92 ± 0.07a	19.97 ± 0.60ab	15.84 ± 0.88a–e
	8	3.92 ± 0.08cd	0.89 ± 0.13a–c	79.67 ± 0.80d	3.15 ± 0.06a–c	6.13 ± 0.50a	1.94 ± 0.05a	20.32 ± 0.80a	15.53 ± 0.87a–e
T_3_	0	4.27 ± 0.07ab	0.55 ± 0.05d–h	80.49 ± 0.73cd	3.07 ± 0.05a–d	6.10 ± 0.28a	1.58 ± 0.09a	19.51 ± 0.73ab	14.98 ± 0.62b–e
	2	4.18 ± 0.05a–c	0.60 ± 0.09c–h	80.94 ± 0.80cd	2.92 ± 0.06b–d	6.05 ± 0.60a	1.61 ± 0.08a	19.43 ± 0.80ab	16.17 ± 0.98a–d
	4	4.13 ± 0.04a–d	0.66 ± 0.12b–h	80.56 ± 0.80cd	3.04 ± 0.04a–d	6.09 ± 0.55a	1.64 ± 0.11a	19.05 ± 0.80ab	15.69 ± 0.82a–e
	6	4.09 ± 0.07a–d	0.80 ± 0.18a–f	80.40 ± 0.76cd	2.96 ± 0.08a–d	6.13 ± 0.75a	1.66 ± 0.15a	19.59 ± 0.76ab	15.13 ± 0.98b–e
	8	3.89 ± 0.08cd	0.88 ± 0.14a–c	79.63 ± 0.95d	2.85 ± 0.06d	6.16 ± 0.20a	1.62 ± 0.14a	20.36 ± 0.95a	14.03 ± 0.90c–e
T_4_	0	4.33 ± 0.10ab	0.51 ± 0.04f–h	80.36 ± 0.28cd	2.96 ± 0.16a–d	6.10 ± 0.26a	1.64 ± 0.90a	19.63 ± 0.28ab	15.11 ± 1.01b–e
	2	4.35 ± 0.27ab	0.57 ± 0.06d–h	80.23 ± 1.20cd	3.10 ± 0.20a–d	6.01 ± 0.24a	1.69 ± 0.11a	19.76 ± 1.20ab	15.76 ± 1.59a–e
	4	4.24 ± 0.25ab	0.70 ± 0.07a–h	81.36 ± 0.29cd	3.08 ± 0.07a–d	6.09 ± 0.37a	1.72 ± 0.14a	18.63 ± 0.29ab	16.55 ± 0.32a–d
	6	4.09 ± 0.04a–d	0.82 ± 0.10a–e	81.60 ± 0.42b–d	3.21 ± 0.19ab	6.06 ± 0.28a	1.74 ± 0.18a	18.39 ± 0.42ab	17.46 ± 1.32ab
	8	3.92 ± 0.03cd	0.98 ± 0.14a	82.60 ± 0.90a–c	3.22 ± 0.15a	6.11 ± 0.35a	1.75 ± 0.16a	17.40 ± 0.90b–d	18.57 ± 1.86a

*Note:* Means with different letters in the same column show significant (*p <* 0.05) differences between treatments and storage period.

The more decreasing trend of pH in date paste added dahi might be due to more activities of indigenous microorganisms due to presence of more nutrients, thereby resulted in more accumulation of acids leading to more acidic conditions. Our results of pH and acidity were in agreement with those of Dias *et al*. (2021) and Hamid and Doosh (2021) who also observed decreasing trend of pH and increasing trend of acidity of yogurt during storage. The presence of date paste might serve as substrate for microorganisms. The increased contents of fat, protein, and ash in dahi supplemented with date paste might be attributed to the presence of these contents in dates. Our results of TS were in agreement with those of Gyawali and Ibrahim (2018) who also observed ~13% of TS in yogurt prepared from cow's milk. The higher moisture contents of dahi prepared from buffalo milk might be due to more TS of buffalo milk. The curd settled from buffalo milk was observed to have good thickness and consistency compared to the curd obtained from cow's milk.

### 3.4 Microbiological Quality of Dahi

**FIGURE 1:**
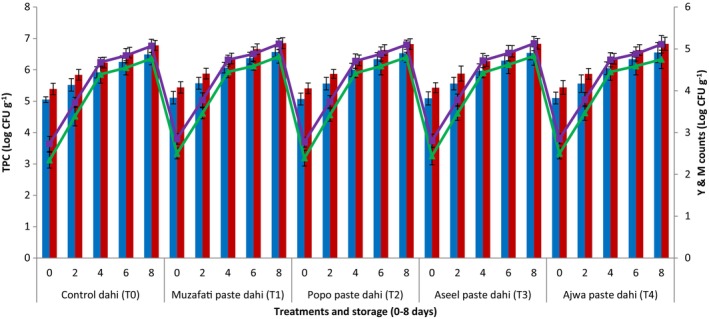
Total plate counts (TPC) (blue bars = dahi from cow's milk, red bars = dahi from buffalo's milk) and yeast and molds (Y&M) (green lines = dahi from cow's milk, purple lines = dahi from buffalo's milk) of dahi supplemented with pastes from different date varieties during storage (0–8 days) at 4°C.

Figure 1 shows the effect of date fruit paste supplementation in dahi on TPC (log_10_) and Y&M (log_10_). The TPC of control dahi (without the addition of date paste) were 5.05 (log_10_) which continued to increase reaching till 6.49 (log_10_) after 8 days of storage. But dahi samples supplemented with date paste showed slightly higher values (~6.55 log_10_) compared to control paneer after 8 days of storage. Generally, it was observed that the results concerning TPC (log_10_) of all treatments were significantly (*p <* 0.05) different and the counts were steadily increased in all treatments of dahi prepared from both cow and buffalo's milk. Similar trend was also observed regarding Y&M (log_10_). The values of Y&M also significantly (*p <* 0.05) increased during storage in all the treatments. All the dahi supplemented with date paste showed more TPC and Y&M values compared to the values of control dahi. The TPC as well as Y&M (log_10_) counts of dahi prepared from buffalo's milk was slightly higher than the dahi made from cow's milk.

Our results of microbial counts were in accordance with the findings of Anjum, Rahman, and Gani (2020) who also observed increasing trend of TPC and Y&M counts of dahi during storage. The higher TPC and Y&M counts of dahi prepared from buffalo milk might be due to presence of more nutrients for the growth of different contaminated microorganisms.

### 3.5 Sensorial Quality of Prepared Dahi

**FIGURE 2:**
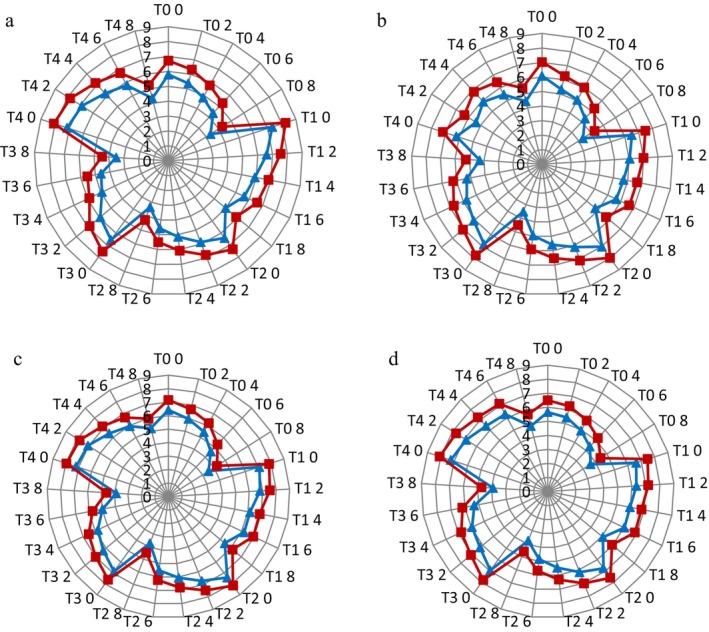
Sensory acceptance scores regarding appearance and color (a, blue line = dahi from cow's milk, red line = dahi from buffalo's milk), flavor (b, blue line = dahi from cow's milk, red line = dahi from buffalo's milk), texture (c, blue line = dahi from cow's milk, red line = dahi from buffalo's milk) and overall acceptability (d, blue line = dahi from cow's milk, red line = dahi from buffalo's milk) of dahi (T_0_ = control, T_1_ = Muzafati paste dahi, T_2_ = Popo paste dahi, T_3_ = Aseel paste dahi, T_4_ = Ajwa paste dahi) supplemented with pastes from different date varieties during storage (0–8 days) at 4°C.

Figure 2a–d shows the effect of date paste on different sensory attributes, that is, appearance and color, flavor, texture, and overall acceptability of dahi prepared from cow’ milk and buffalo's milk. The flavor score of cow's milk control dahi (T_0_, without the addition of date paste) started at 5.65 which continued to decrease reaching till 3.62 after 8 days of storage (Figure 2b). Similarly, the texture score of cow's milk control dahi (without the addition of date paste) started at 6.09 which continued to decrease reaching till 3.34 after 8 days of storage (Figure 2c). But, dahi supplemented with date paste (T_1_–T_4_) had significantly (*p <* 0.05) higher sensory scores than control treatment (dahi) at each storage period. Generally, all freshly prepared dahi either control or supplemented with date paste had higher sensory scores for all the sensory attributes. It was observed that the dahi supplemented with Ajwa date paste obtained the highest score among all the treatments. The sensory score of all the attributes decreased significantly (*p <* 0.05) during storage.

Figure 2a shows the effect of date paste addition on appearance and color of dahi from both cow's and buffalo's milk. The score of appearance and color of freshly prepared control dahi from buffalo's milk (6.72) was higher compared to the score obtained by respective dahi made from cow's milk (5.79) which might be due to more settled curd from buffalo's milk. The thick consistency of buffalo's milk may contribute to good appearance and color of dahi. Similar trend was observed regarding flavor of dahi. The taste of dahi prepared from buffalo's milk was good compared to cow's milk dahi. The texture score of freshly prepared dahi from buffalo's milk (7.04) was also higher than respective dahi made from cow's milk (6.09). The score of overall acceptability of dahi prepared from buffalo's milk was also more compared to the score obtained by dahi made from cow's milk (Figure 2d).

Among all the treatments (dahi from cow's and buffalo's milk dahi), there was a decreasing trend for all the sensory attributes during storage. The sensory attributes also illustrate quality characteristics of prepared dahi. The higher scores of appearance and color as well as texture of dahi prepared from buffalo milk might be due to more settled curd from buffalo milk. The thick consistency of buffalo milk may contribute to good appearance and color of dahi. The higher contents of protein and TS in buffalo milk might contribute to good consistency of dahi. Our results were in agreement with the findings of Korkmaz, Bilici, and Korkmaz (2021) who also observed decreasing sensory scores of yogurt supplemented with maca (*Lepidium meyenii*) powder and propolis extract. Similarly, Hamid and Doosh (2021) also reported decreasing trend of sensory scores of yogurt.

### 3.6 Syneresis (%) and WHC (%) of Dahi

**FIGURE 3:**
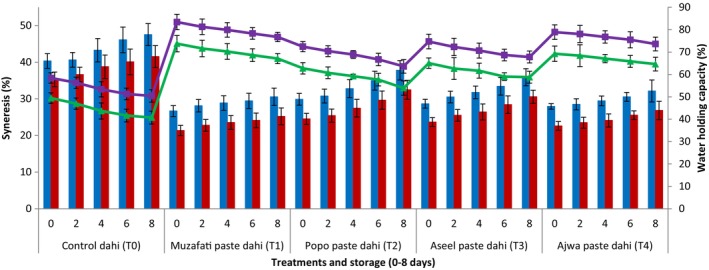
Syneresis (%) (blue bars = dahi from cow's milk, red bars = dahi from buffalo's milk) and water holding capacity (%) (green lines = dahi from cow's milk, purple lines = dahi from buffalo's milk) of dahi supplemented with pastes from different date varieties during storage (0–8 days) at 4°C.

Figure 3 shows the effect of date fruit paste addition on syneresis and WHC of dahi samples. The syneresis of freshly prepared cow's milk control dahi (without the addition of date paste) started with 40.33 (%) which continued to increase reaching till 47.54 (%) after 8 days of storage. The dahi supplemented with date paste had significantly (*p <* 0.05) lower syneresis than control treatment (dahi) at each storage period. Generally, all freshly prepared dahi either control or supplemented with date paste had lower values of syneresis which significantly (*p <* 0.05) increased during subsequent storage.

The WHC (%) of freshly prepared cow's milk control dahi (without the addition of date paste) started with 49.43 (%) which continued to increase reaching till 40.73 (%) after 8 days of storage. The dahi supplemented with date paste had higher WHC (%) than control treatment (dahi) at each storage period. On the other hand, all freshly prepared dahi either control or supplemented with date paste had higher values of WHC (%) which significantly (*p <* 0.05) decreased during subsequent storage.

The syneresis of dahi prepared from buffalo's milk was lower than dahi made from cow's milk while WHC (%) of dahi prepared from buffalo's milk was higher than dahi made from cow's milk. Therefore, dahi prepared from buffalo's milk was considered better compared to cow's milk dahi due to less syneresis and more WHC. Generally, all the treatments (dahi from each cow's and buffalo's milk dahi) showed increasing trend of syneresis (%) during storage whereas WHC (%) depicted decreasing trend.

The lower syneresis of dahi supplemented with date paste might be due to more consistent curd after supplementation of date pastes. The moisture contents were bound more in the matrix of dahi, thereby leading to less syneresis. Our results regarding WHC (%) were consistent to the findings of Ghaderi‐Ghahfarokhi *et al*. (2021) and Costa *et al*. (2022) who also observed decreasing trend of WHC (%) during subsequent storage. In another study, WHC (%) of yogurt made from buffalo milk observed as 50% (Atallah, Morsy, and Gemiel 2020) which was consistent to the findings of the present study. The higher WHC (%) of dahi supplemented with date paste might be attributed to more consistent curd after supplementation of date pastes. The moisture contents were bound more in the matrix of dahi, thereby leading to more WHC (%). The lower syneresis and higher WHC (%) of dahi prepared from buffalo milk might be due to higher contents of protein and some other nutrients.

### 3.7 Antioxidant Potential of Prepared Dahi

**FIGURE 4:**
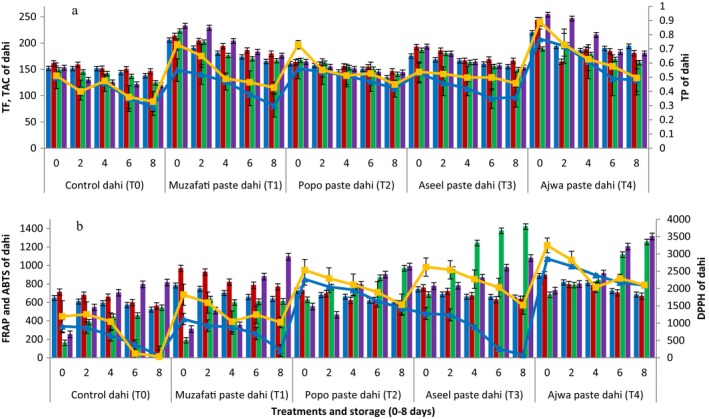
Total phenolics (TP, mg GAE/g) (blue lines = dahi from cow's milk, orange lines = dahi from buffalo's milk), total flavonoids (TF, mg CE/100 g) (blue bars = dahi from cow's milk, red bars = dahi from buffalo's milk), total antioxidant activity (TAC) (mg AAE/100 g) (green bars = dahi from cow's milk, purple bars = dahi from buffalo's milk), ferric reducing antioxidant power (FRAP, mg TE/100 g) (blue bars = dahi from cow's milk, red bars = dahi from buffalo's milk), ABTS radical scavenging activity (μM AAE/g) green bars = dahi from cow's milk, purple bars = dahi from buffalo's milk), and DPPH radical scavenging activity (μM AAE/g) (blue lines = dahi from cow's milk, orange lines = dahi from buffalo's milk) of dahi supplemented with pastes from different date varieties during storage (0–8 days) at 4°C.

Figure 4a,b shows the results concerning antioxidant potential of dahi prepared in the present study. The control dahi (T_0_, without the addition of date paste) showed significantly (*p <* 0.05) lower values in all these assays compared to all other treatments at all the stages during storage period (0–8 days). In general, freshly prepared as well as 2 and 4 days stored dahi supplemented with different date pastes presented significantly (*p <* 0.05) higher TP, TF, TAC, ABTS/DPPH radical scavenging activities and FRAP values in all the treatments. The TF values of freshly prepared control dahi (without the addition of date paste) from cow's milk were 151.83 mg CE/100 g which were significantly (*p <* 0.05) lower than all the dahi supplemented with date pastes. The TAC values of freshly prepared control dahi from cow's milk were 149.10 mg AAE/100 g, which were significantly (*p <* 0.05) lower than all the dahi supplemented with date pastes. Similarly, FRAP values of freshly prepared control dahi from cow's milk were 711.66 μg AAE/g which were significantly (*p <* 0.05) lower than all the dahi supplemented with date pastes. The ABTS radical scavenging activity values of freshly prepared control dahi from cow's milk were 162.69 μM AAE/g which were significantly (*p <* 0.05) lower than all freshly made cow's milk dahi supplemented with date pastes. The DPPH radical scavenging activity values of freshly prepared control dahi from cow's milk were 910 μM AAE/g which were significantly (*p <* 0.05) lower than all cow's milk dahi supplemented with date pastes. It was also observed that dahi supplemented with Ajwa paste (T_4_) performed the best with regard to TP, TF, ABTS/DPPH radical scavenging activity and TAC.

The values concerning TP, TF, TAC, ABTS/DPPH radical scavenging activities and FRAP of dahi prepared from buffalo were slightly higher than the respective values obtained by dahi made from cow's milk.

Our results of TP contents of dahi were in agreement to the investigations of Frumento et al. (2013) who observed more or less same values (~1 mg GAE/g) as observed in the present study. In another study, TP contents of yogurt made from buffalo milk were reported as 0.15 mg GAE/g (Atallah, Morsy, and Gemiel 2020). Those reported values were lower than the values observed in the present study. The more FRAP values of dahi samples supplemented with date pastes depicted the presence of more TP and TF of date pastes. The FRAP of date paste may exhibit potent antioxidant activity due to presence of antioxidants (phenolics and flavonoids). The higher values of both ABTS as well as DPPH radical scavenging activities of date pastes supplemented dahi also confirmed the presence of enormous quantities of TP and TF in date fruits. The phenolic compounds present in dates include gallic acid, vanillic acid, caffeic acid, syringic acid, *p*‐coumaric acid, ferulic acid, and sinapic acid whereas hesperidin, luteolin, quercetin, kaempferol, and isorhamnetin are flavonoids (Hinkaew *et al*. 2021). The slightly higher values concerning TP, TF, TAC, ABTS/DPPH radical scavenging activities and FRAP of dahi prepared from buffalo might be due to presence of more proteins and other bioactive compounds in buffalo milk compared to cow's milk.

Table 6 shows Pearson's correlation coefficients between the investigated antioxidant capacity parameters (DPPH, ABTS, RP, TAC, TF, and TP) of buffalo's milk dahi and cow's milk dahi. The ABTS showed significant and negatively high correlations with FRAP, TAC, TF, and TP of buffalo's as well as cow's milk dahi. The DPPH showed poor negative correlations with FRAP, TAC, TF, and TP of buffalo's as well as cow's milk dahi. The FRAP showed significant and strong positive correlations with TAC, TF, and TP of buffalo's as well as cow's milk dahi. The TAC showed significant and positively high correlations with TF and TP of buffalo's as well as cow's milk dahi. There was positive correlation between TF and TP of buffalo's as well as cow's milk dahi. There was very poor and non‐significant correlation existed between ABTS and DPPH. The results suggested that both phenolic contents and flavonoid contents may be considered as the most important contributors to the total antioxidant capacity as well as ferric reducing antioxidant power.

**TABLE 6:** Pearson's correlation coefficient (*R*) (*p* value) between the investigated antioxidant capacity parameters (DPPH radical scavenging activity, ABTS radical scavenging activity, ferric reducing antioxidant power (FRAP), total antioxidant activity (TAC), total flavonoids (TF) and total phenolics (TP)) of buffalo's milk dahi and cow's milk dahi.

Parameters	ABTS	DPPH	FRAP	TAC	TF
Buffalo's milk dahi					
DPPH	0.1271 (0.2773)				
FRAP	−0.7313 (0.0000)	−0.1656 (0.1557)			
TAC	−0.7191 (0.0000)	−0.398 (0.0004)	0.8672 (0.0000)		
TF	−0.6215 (0.0000)	−0.3100 (0.0068)	0.8513 (0.0000)	0.8396 (0.0000)	
TP	−0.6326 (0.0000)	−0.0364 (0.7568)	0.6984 (0.0000)	0.7453 (0.0000)	0.6605 (0.0000)
Cow's milk dahi					
DPPH	0.1082 (0.3554)				
FRAP	−0.6520 (0.0000)	−0.3449 (0.0024)			
TAC	−0.6593 (0.0000)	−0.3920 (0.0005)	0.8435 (0.0000)		
TF	−0.7172 (0.0000)	−0.5405 (0.0000)	0.8911 (0.0000)	0.8622 (0.0000)	
TP	−0.5569 (0.0000)	−0.0538 (0.6465)	0.8447 (0.0000)	0.5988 (0.0000)	0.6572 (0.0000)

### 3.8 ACE Inhibitory Activity of Prepared Dahi

**FIGURE 5:**
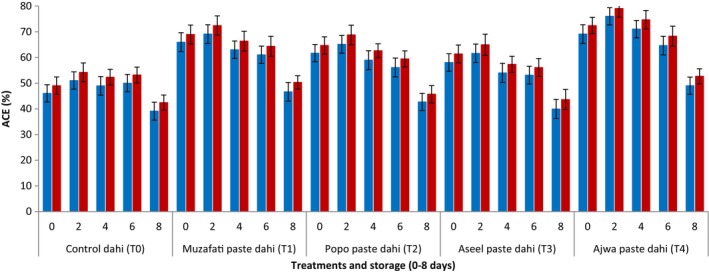
ACE (%) inhibition (blue bars = dahi from cow's milk, red bars = dahi from buffalo's milk) of dahi supplemented with pastes from different date varieties during storage (0–8 days) at 4°C.

Figure 5 shows the effect of date fruit paste addition on ACE inhibitory activity of dahi samples. The control dahi (without the addition of date paste) prepared from cow's milk showed 46.3% of ACE inhibition in the start which continued to decrease reaching till 39.10% after 8 days of storage. The results showed that all the dahi supplemented with date paste had significantly (*p <* 0.05) higher ACE inhibition (%) as compared to control treatment (dahi). In most of the cases, the values increased significantly (*p <* 0.05) until 4 days of storage but afterwards started to decrease in subsequent storage period. The maximum values (69.10%) were shown by cow's milk dahi supplemented with Ajwa paste followed by cow's milk dahi supplemented with Muzafati paste (65.93%). The ACE inhibition (%) of dahi prepared from buffalo's milk was slightly higher than the respective dahi samples made from cow's milk.

The higher ACE inhibition (%) of dahi supplemented with date pastes than control dahi might be due to presence of more bioactive compounds from date pastes. A study showed that extracts of dates exhibited significant ACE inhibitory effects (Obode, Adebayo, and Li 2020). They concluded that such kind of ACE inhibition could possibly lead to the antihypertensive effects of dates. Further, they emphasized that dates may serve as an alternative therapy for hypertension management in order to avoid the adverse effects associated with antihypertensive drugs. Al‐Dashti *et al*. (2021) reported that date fruits being rich source of polyphenols, certain micronutrients, and dietary fiber, can impact vascular health by modulating plasma lipid levels including triglycerides and cholesterol, indices of oxidative stress and inflammation. Moreover, date fruit contains the highest concentrations of potassium compared to other minerals which is very helpful in regulation of blood pressure (Bentrad and Hamida‐Ferhat 2020). The slightly higher ACE inhibitory activity of dahi prepared from buffalo’ milk might be due to presence of more proteins and other bioactive compounds in buffalo milk compared to cow's milk.

The desired properties of the investigated dahi were influenced by date fruit pastes, type of milk, and storage. For instance, sensory acceptability of dahi prepared from buffalo's milk was more compared to the scores obtained by dahi made from cow's milk. All the prepared dahi supplemented with date pastes were liked by assessors. All the prepared dahi supplemented with date paste had lower values of syneresis and higher WHC (%). The syneresis of dahi prepared from buffalo's milk was lower than dahi made from cow's milk while WHC (%) of dahi prepared from buffalo's milk was higher than dahi made from cow's milk. The values concerning TP, TF, TAC, ABTS/DPPH radical scavenging activities and FRAP of dahi prepared from buffalo's milk were higher than from cow's milk. It was also observed that dahi supplemented with Ajwa paste (T_4_) performed the best with regard to TP, TF, ABTS/DPPH radical scavenging activity and TAC. The ACE inhibitory activity of dahi prepared from buffalo's milk was slightly higher than dahi prepared from cow's milk.

## 4 Conclusion

Generally, all the prepared dahi supplemented with date pastes were liked by assessors. The syneresis of dahi prepared from buffalo's milk was lower than dahi made from cow's milk while WHC (%) of dahi prepared from buffalo's milk was higher than dahi made from cow's milk. The values concerning TP, TF, TAC, ABTS/DPPH radical scavenging activities, FRAP, and ACE inhibitory activity of dahi prepared from buffalo's milk were higher than from cow's milk. It was also observed that dahi supplemented with Ajwa paste (T_4_) performed the best with regard to TP, TF, ABTS/DPPH radical scavenging activity, TAC, and ACE inhibitory activity.

Owing to the presence of considerable quantities of antioxidants and other compounds in low‐fat dahi supplemented with date fruit paste, this product is recommended to the dairy industry for marketing to consumers who are suffering from hypertension and obesity.

## Author Contributions

**Tahir Mahmood Qureshi:** conceptualization (equal), data curation (equal), investigation (equal), methodology (equal), project administration (equal), supervision (equal), validation (equal), writing – original draft (equal), writing – review and editing (equal). **Muhammad Nadeem:** data curation (equal), software (equal), validation (equal), writing – review and editing (equal). **Ghulam Muhammad:** data curation (equal), formal analysis (equal). **Waseem Abbas:** formal analysis (equal). **Kashif Akram:** writing – review and editing (equal). **Majid Hussain:** writing – review and editing (equal). **Zahid Manzoor:** writing – review and editing (equal). **Salam A. Ibrahim:** funding acquisition (equal), visualization (equal), writing – review and editing (equal).

## Conflicts of Interest

The authors declare no conflicts of interest.

## Data Availability

Data are contained within the article.
